# Behaviours and psychological symptoms of childhood dementia: two cases of psychosocial interventions

**DOI:** 10.1177/26323524241273492

**Published:** 2024-09-06

**Authors:** Mustafa Atee, Ineka Whiteman, Rebecca Lloyd, Thomas Morris

**Affiliations:** The Dementia Centre, HammondCare, Level 2, 302 Selby Street Nth, Osborne Park, WA 6017, Australia; Sydney Pharmacy School, Faculty of Medicine and Health, The University of Sydney, Sydney, NSW, Australia; Curtin Medical School, Faculty of Health Sciences, Curtin University, Bentley, WA, Australia; Centre for Research in Aged Care, School of Nursing and Midwifery, Edith Cowan University, Joondalup, WA, Australia; Batten Disease Support & Research Association (BDSRA) Australia, Brisbane, QLD, Australia; BDSRA Foundation, Columbus, OH, USA; Beyond Batten Disease Foundation, Austin, TX, USA; The Dementia Centre, HammondCare, Osborne Park, WA, Australia; The Dementia Centre, HammondCare, St Leonards, NSW, Australia; Sydney School of Public Health, Faculty of Medicine and Health, The University of Sydney, Sydney, NSW, Australia; Thomas Morris is also affiliated with Faculty of Health, University of Canberra, Bruce, ACT, Australia

**Keywords:** Batten disease, behaviours and psychological symptoms of dementia, case report, childhood dementia, dementia behaviour support programs, neuropsychiatric symptoms, psychosocial interventions, Sanfilippo syndrome

## Abstract

Childhood dementias are a group of rare, fatal neurodegenerative disorders, characterised by global cognitive decline, loss of previously acquired developmental skills and behaviours and psychological symptoms of dementia (BPSD). Batten disease, or neuronal ceroid lipofuscinosis, and Sanfilippo syndrome, or mucopolysaccharidosis type III, are two of the more common forms of childhood dementia disorders worldwide. While psychosocial interventions are the best available therapeutic approach for BPSD management in adult-onset dementia, there is very limited literature or clinical experience in the context of childhood dementia. To address this gap, we conducted a descriptive case analysis of BPSD profiles, associated contributing factors and targeted psychosocial interventions in two cases with childhood dementia disorders (Sanfilippo syndrome and CLN3 (juvenile onset) Batten disease) who were referred to Dementia Support Australia, a national dementia behaviour support service in Australia. Primary BPSD identified in these disorders included physical and verbal aggression and irritability/lability. In these cases, contributing factors to the development of BPSD were not monolithic, encompassing pain, caregiver’s approach and over or under-stimulation. Improvement in BPSD were observed using the Neuropsychiatric Inventory-Quesionnaire and globally noted as per the qualitative feedback reported by family and caregivers. Person-centred, multimodal psychosocial interventions were recognised as effective therapies in resolving BPSD in these cases. In conclusion, the case studies described the nature and presentation of BPSD in two common forms of childhood dementia and demonstrated the potential benefits of person-centred psychosocial interventions (delivered through national dementia-specific support programs) in alleviating BPSD such as irritability and aggression in these disorders.

## Introduction

Childhood dementia (CD) is a group of disorders defined by global, progressive neurocognitive decline during childhood or adolescence, usually following a period of typical development.^1^ The enduring and progressive loss of previously acquired cognitive function is distinct from developmental delay or intellectual disability which, by contrast, are characterised by slow albeit increasing developmental trajectories.^1,2^ Caused by more than 70 individual rare genetic diseases, CD collectively is estimated to affect more than 700,000 people worldwide, including around 2200 Australians.^3,4^

Each year, around 48,000 children and young people globally and more than 90 in Australia die from a form of CD.^4^ Most deaths occur in individuals under 18 years. Presently, less than 5% of CD have a treatment, and many of those are limited to symptom management only or, at best, may slow disease progression. While CD affects a relatively small number of individuals, health economics studies suggest that the resulting burden to families, the economy, and society at large is significant, and has been estimated to cost the Australian economy and healthcare system over AU$389 million annually.^3,4^

Batten disease and Sanfilippo syndrome are two of the more common forms of CD.^1,3–6^ Both conditions are lysosomal storage disorders, a group of genetic entities characterised by the dysfunction of lysosomal proteins, leading to persistent aggregation of inefficiently recycled intracellular waste and eventually cell death, especially within the brain.^7^ Batten disease and Sanfilippo syndrome share broadly similar clinical phenotypes with affected children experiencing loss of cognitive skills, decline in language and speech, seizures, loss of mobility, movement disorders and premature death.^5,8^

Broadly, behaviours and psychological symptoms of dementia (BPSD) are defined as cardinal non-cognitive symptoms in all types of dementia that often occur in response to organic aetiology, unmet need and/or environmental factors.^9^ Approximately 90% of individuals with adult-onset dementia exhibit at least one BPSD.^10^ Examples of BPSD in CD include agitation and aggression, attention problems, social and emotional dysfunctions, unresponsiveness and sleep disturbances.^11–15^ Psychotic symptoms such as visual hallucinations, delusions and obsessive-compulsive symptoms are also common.^16^ Research in adult-onset dementias indicates that BPSD are associated with a person’s impaired quality of life, significant distress, frequent hospitalisation, premature institutionalisation and increased caregiver distress.^15,17^

Similarly, in the context of CD, studies show that the devastating disease course and associated BPSD are salient and burdensome, conferring a significant detrimental impact on quality of life for the affected individual, their caregivers and the entire family unit.^18,19^ Oscillations between mood and behaviour, including physical and verbal aggression, hyperactivity and severe emotional dysregulation, are reportedly associated with increased distress and frustration for caregivers, siblings and other family members, increasing need for vigilance and reluctance to partake in activities outside of the home, leading to caregiver isolation and physical and emotional burden.^20^

### Sanfilippo syndrome

Sanfilippo syndrome, or mucopolysaccharidosis type III (MPS III), is a group of conditions comprised of MPS IIIA, B, C, D, with type A being most common.^8,12,21^ The estimated prevalence of MPS III is between 0.3 and 4.1 cases for every 100,000 live births.^8^ The clinical course of Sanfilippo syndrome is characterised by three phases. Phase I typically starts with developmental and language delay between 1 and 4 years of age. At around 3–4 years, the second phase is typically characterised by severe behavioural problems, hyperactivity unresponsive to medication, verbal and physical aggression, sleep abnormalities, progressive mental deterioration and severe dementia. In the third and end phase of Sanfilippo syndrome, behavioural problems gradually decrease with emergence of motor retardation, swallowing difficulties, seizures and spasticity.^12,21^ Death typically occurs by the early 30s.^12,21^ Caregiver burden is cumulative and significant throughout the lifespan, as caregivers manage increasing symptom load and severity.^22,23^

### Batten disease

Batten disease is the common name for a family of conditions known as neuronal ceroid lipofuscinoses, or NCLs, comprised of 13 different subtypes (CLN1 disease through to CLN14 disease; there is no CLN9 disease).^24,25^ CLN3 disease (juvenile onset) and CLN2 disease (late infantile onset) are the most common forms, together comprising more than half of all Batten disease cases worldwide.^25^ With an estimated prevalence of 1–13 per 100,000 live births, Batten disease subtypes share a broadly similar clinical presentation including progressive visual failure, cognitive decline, behavioural changes, language delays and loss of speech, seizures, movement disorders and loss of motor control, sleep disturbance, severely reduced quality of life and premature death in late childhood to early adulthood.^5,26–28^

Unlike some CDs, BPSD are considered primary symptoms of Batten disease.^29,30^ Behavioural changes, particularly in CLN3 disease, include irritability, verbal and physical aggression, severe anxiety and fearfulness, obsessive-compulsive-like behaviours, depressed mood, perseverance and vocalisation.^31^ In CLN3 disease, symptoms tend to increase through childhood and middle teen years, and then decrease towards end stages of the disease, except anxiety and fearful behaviours which typically increase during the latter stages of CLN3 disease.^16,31,32^

#### Research rationale and objectives

The current international guidelines place person-centred psychosocial interventions as the best available therapeutic option for BPSD management in the adult-care setting.^15,33,34^ Psychosocial interventions in dementia care aim to improve the physical, psychological and/or social functioning of individuals. This includes physical and psychological well-being, cognitive function, interpersonal relationships, behaviour and quality of life. Psychosocial interventions can be equally or more effective than pharmacological interventions and complement holistic treatment by supporting family and caregivers through education and support programs.^35–38^ Person-centred approaches place the individual at the forefront of service, focusing on care, support and treatment that are most important to the complex needs of the individual, their family and caregivers.^39^

In Australia, these interventions are offered nationally through specialised programs by Dementia Support Australia (DSA).^9^ While the literature is abundant with examples of psychosocial interventions for younger-onset and older-onset dementia, little attention has been given to these interventions in the context of childhood-onset dementia. Here, we report two CD cases referred to DSA for BPSD support and describe the person-centred psychosocial interventions implemented and qualitative outcomes achieved.^35,36,40^

## Context

A descriptive case analysis of BPSD profiles, contributing factors and psychosocial interventions was conducted for two individuals with CD who were referred by their caregivers to DSA, a national and federally funded multidisciplinary dementia-specific behaviour support service in Australia.^41^ This involves comprehensive BPSD assessments and targeted support and recommendation strategies.^41^

**Figure 1.**
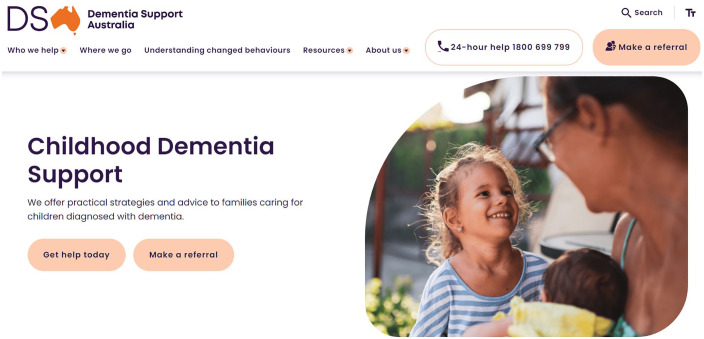
A screenshot of the Childhood Dementia Support website created by Dementia Support Australia (DSA).

DSA provides multimodal person-centred non-pharmacological psychosocial interventions free-of-charge to people living with BPSD, and their caregivers.^41^ This national support service delivers these interventions primarily through two programs: the Dementia Behaviour Management Advisory Service for mild-to-moderate BPSD, and the Severe Behaviour Response Teams for severe forms of BPSD. These programs operate within a large multidisciplinary team that comprises dementia consultants, geriatricians, old age psychiatry doctors, psychogeriatricians and support staff. DSA consultants encompass a wide range of dementia (including CD) and aged care expertise, such as nursing, occupational therapy, physiotherapy and diversional therapy. All consultants receive training on dementia.^41^ Further, a specialised CD support program (Figure 1) is available as part of DSA service with training designed and delivered in conjunction with the Batten Disease Support & Research Association (BDSRA) Australia and the Childhood Dementia Initiative.

DSA referrals are received routinely via the DSA website, a dedicated phone line, or in person, most commonly via formal caregivers or aged care staff in a residential setting and informal caregivers in a community setting.^41^

Prior to support, each referral is triaged and assessed for eligibility. Eligibility criteria for DSA support include individuals with a confirmed or probable diagnosis of dementia, including any of the identified CDs, and BPSD that affect the referred person and/or their caregiver.^42^ For eligible referrals, an extensive onsite assessment of medico-social history and living environment is conducted to identify potential factors contributing to the development of BPSD. Assessment data, including clinical presentation and socio-demographic information, are collected by trained DSA staff and entered into a dedicated, secure database.^41,42^ As part of the assessment, two clinical validated tools are administered routinely by DSA: the Neuropsychiatric Inventory (NPI) and PainChek^®^ (Table 1).^43–48^

**Table 1.** Description of clinical instruments used for the cases.

Instrument	Description
Neuropsychiatric Inventory – Questionnaire (NPI-Q)	A valid and reliable informant-based tool that evaluates 12 domains of neuropsychiatric symptoms or BPSD: aberrant motor behaviour, agitation/aggression, anxiety, apathy/indifference, appetite and eating, delusions, depression/dysphoria, disinhibition, elation/euphoria, hallucinations, irritability/lability and night-time behaviour.^43^ The NPI-Q rates each domain on its presence (yes/no) and severity (‘mild’ [1], ‘moderate’ [2], ‘severe’ [3]). Further, NPI-Q reports the caregiver distress/disruptiveness associated with each of the 12 domains with ratings from (‘not at all’ [0], ‘minimally’ [1], ‘mildly’ [2], ‘moderately’ [3], ‘severely’ [4], ‘extremely’ [5]). The total caregiver distress or caregiver disruptiveness scores can be calculated separately by adding the distress/disruptiveness scores of all 12 domains. Higher NPI-Q scores indicate more severe and more distressing behaviours.^43^
PainChek^®^	A validated artificial intelligence-enabled pain assessment tool in the form of a point-of-care smart device application (app) for non-verbal people living with dementia, which is registered as a medical device by Australia’s Therapeutic Goods Administration, Health Canada, Singapore Health Sciences Authority and European Conformity.^44–46^ The tool has 42-items covering six domains (Face (9 items), Voice (9 items), Movement (7 items), Behaviour (7 items), Activity (4 items) and Body (6 items)). Items are rated on a binary level (Yes = present, No = absent) and each item is provided with a clear definition to improve interrater consistency.^46,47^ Pain is scored if PainChek® total score is ⩾7 and absent if the total score is <7.^48^

Psychosocial interventions recommended by DSA consultants may include support strategies, capacity building (for affected individuals and/or their caregivers) and brokerage. The latter refers to any resources or services, including devices, products, therapy or support staff, that aim to improve quality of life and deliver meaningful engagement for individuals. DSA focus on implementing best practice and evidence-based care that aims to reduce behaviours negatively impacting the referred individual and their caregivers.^41^ Examples of brokerage items include (but are not limited to) allied health services such as physiotherapy or occupational therapy, communication and assistive technologies such as smart tablets, music therapy, simulated pets, sensory items and entertainment. For CD referrals, consultants provide additional educational support and guidance to the referrals’ broader multidisciplinary support team including support workers, allied health and education providers to enable a better understanding of the condition, dementia-related behaviours and to aid in the optimal implementation of DSA recommendations.

Further information on DSA programs and their respective models of care can be found in Macfarlane et al.^41^

To guide our reporting and to ensure data completeness and transparency, we used the case report (CARE) guidelines checklist (Supplemental File) for the present study.^49^

To maintain anonymity, pseudonyms were used throughout this paper.^50^

## Cases

We selected two cases of CD in their twenties supported by DSA during 2020–2021, as they were representative of the underlying disorders and information-rich, enabling an in-depth analysis of BPSD and associated contributing factors, and exemplary of psychosocial strategies used by dementia-specific consultants to address BPSD in this population. The cases (summarised in Table 2) had an average length of support of 103 days. Table 3 provides definitions for the clinical terms used in these cases.

**Table 2.** A summary of neuropsychiatric assessments at baseline (intake) and follow-up (discharge), BPSD triggers and main psychosocial strategies used to support the childhood dementia cases reported in the study.

Case	Childhood dementia diagnosis	Current medications	Baseline neuropsychiatric assessment		BPSD triggers and applied interventions		Follow-up neuropsychiatric assessment
			Primary BPSD that led to referral*	NPI-Q	Key contributing factor(s)*	Main psychosocial strategies^#^	NPI-Q
Ava (female)	Sanfilippo syndrome	• Quetiapine tablets for aggression and related behaviours • Cannabis oil for agitation management • Atropine drops for hypersalivation • Paracetamol tablets (PRN) for pain relief	Verbal aggression	*Total domain score*: 6 *Total distress score*: 30 *Total severity score*: 18	• Family dynamics • Caregiver approach • Environment • Over stimulation • Habits/routines • Pain	*Sensory stimulation* • Movie posters • Activity blankets • Pillows • Small, colour-changing balls *Music engagement* • Smart tablet • Headphones • Cue cards *Psychosocial education*	*Total domain score*: 3 *Total distress score*: 3 *Total severity score*: 3
Bianca (female)	Batten’s disease CLN3	• Sodium valproate tablets as mood stabiliser • Olanzapine tablets for behaviours • Melatonin prolonged release tablets for sleep • Mirtazapine tablets for anxiety and depression • Macrogol (PRN) for constipation relief • Paracetamol tablets (PRN) for pain relief	Irritability/lability	*Total domain score*: 6 *Total distress score*: 11 *Total severity score*: 10	• Pain • Loneliness/boredom • environmental factors Relocation stress • Caregiver approach	*Sensory stimulation* • Velcro game • Yarn pack *Assistive technology*• Cloud-based voice service device • Mixing bowl stabiliser *Pet therapy* • Robotic dog • Plush dog *Psychosocial education*	*Total domain score*: 2 *Total distress score*: 8 *Total severity score*: 6

* Primary BPSD observed and key contributing factors identified by DSA consultants.

# Main psychosocial strategies recommended by DSA consultants. These include psychosocial interventions, recommendations, resources and brokerage items.

BPSD, behaviours and psychological symptoms of dementia; DSA, Dementia Support Australia; NPI-Q, Neuropsychiatric Inventory – Questionnaire; PRN, ‘pro re nata’ = administration as needed.

**Table 3.** Clinical terminology used in the manuscript.

Clinical term	Definition
Dysarthria	A weakness in the muscles used for speech which can cause slowed or slurred speech.^51^
Echolalia	The meaningless repetition of vocalisations.^52^
Hyperacusis	A reduced tolerance and increased sensitivity to sound.^53^
Myalgia	Muscle aches and pains that can be localised or widespread.^54^
Oropharyngeal Dysphagia	Swallowing problems occurring in the mouth and/or throat as a product of abnormalities of muscles, nerves or structures.^55^
Scoliosis	An abnormal lateral curvature of the spine.^56^

### Case 1: Ava

#### Background

Ava was a female with Sanfilippo syndrome, who was residing with her parents. She was receiving ongoing support from government-supported disability care, in addition to specialist care. Overnight respite support was provided fortnightly.

Ava was non-verbal (except for the word ‘no’ and nonsensical vocalisations). Her parents reported a significant deterioration in her receptive language and cognitive abilities over the past 12 months. Her mobility and dexterity were also declining, and she had vision impairment, particularly in dim light conditions.

#### Clinical findings

Ava was referred to DSA by her caregivers who sought support strategies for increasing verbal aggression as the primary behaviour of concern. Six probable contributing factors were identified including overstimulation, caregiver approach, changes in habits/routines, environmental factors and pain.

##### Behaviour and communication

At periods throughout the day, particularly upon arriving home after outings, Ava would scream the word ‘no’ repeatedly for 10–15 min. This daily echolalic behaviour had been increasing over a 12-month period. Generally, she responded well to non-verbal communication such as hugs, touch or a smile. The consultant noted episodes of repetitive physical aggression directed towards her parents. For example, immediately after Ava hugged her parents, she hit, cried and screamed at them. In recent months, she appeared to enjoy her outings less and would often become overwhelmed when surrounded by too many people. Ava was also observed to respond with frustration when asked multiple consecutive questions.

##### Activities of daily living

Ava was receiving activities of daily living (ADLs) assistance from her parents, with the support of a female agency caregiver. Resistance to personal care made showering difficult as Ava would grab onto door frames and become rigid. Once she was in the shower however, she would appear relaxed.

Recently, Ava appeared to have an increasing fear of mobilisation. She was still able to walk with assistance on flat surfaces but would constantly look down and become confused and unsteady when walking on surfaces with colour or different textures. Given her declining mobility, a wheelchair was also arranged for Ava’s use as required.

Ava’s parents noted she had not self-reported any pain.

#### Diagnostic assessment

The consultant recorded severe behaviours (Table 2) and severe pain (PainChek^®^ score = 29) for Ava.

#### Therapeutic interventions

##### Reducing overstimulation

To prevent or minimise agitation and anxiety, it was recommended that daily activities be tailored to Ava’s current level of cognition including the provision of one-on-one activities and avoidance of crowded, loud environments. In the event of Ava becoming agitated, it was advised to temporarily pause the activity, offer reassurance and provide her with sufficient time and a quiet space to process her surroundings and emotions.

##### Establishing routine, and validation, reassurance and relaxation therapies

To address Ava’s echolalic vocalisations, the recommendation was to establish a new routine to assist self-regulation while offering reassurance, validation and opportunities for relaxation. A diversional therapist was recommended to help with further tips to tailor daily outing activities for Ava’s current needs.

The family was advised to collaborate with Ava’s caregivers to identify potential triggers for her agitation and anxiety during the transition into the home. Transitions may, for example, be too rapid for Ava to cognitively process, or may cause her to feel unsettled and off-balance when walking through a dark garage or uneven surfaces between the car and house. Ava’s caregiver should remain with her, guide her quietly and gently out of the car to the house without other caregivers or family members approaching to greet her and potentially overwhelm her. Ava should be given time to process these transitions and change in surroundings, and be provided with emotional support, acknowledgement and verbal and physical reassurance.

A recommendation was made for Ava to spend 10 min in a designated ‘private room’ upon entering the home to engage in quiet, pleasurable activities such as watching a favourite film or listening to music. The room should be designed for a comfortable temperature, minimal noise, soft flooring and relevant brokered items to assist with relaxation and her transitions.

##### Sensory engagement

As Ava enjoyed looking at colour, a range of items were brokered for her home, including posters from Ava’s favourite movies, soft touch and glow-in-the-dark fleece and activity/sensory-stimulating blankets, pillows, and a smart tablet including headphones for music engagement. Small colour-changing balls were supplied for visual and tactile stimulation.

##### Communication techniques

The consultant explained that it may be challenging for Ava to understand verbal instructions and engage in ‘conversation’ as she experiences cognitive and communicative changes. A consistent communication approach was suggested. It was observed that some caregivers would communicate with Ava using simple, short, guided instructions in a slow and soft voice. Ava appeared to be much more settled and less verbally aggressive when this approach was used.

Body language and tone of voice should serve to reinforce the message being communicated. As Ava likes to laugh, humour and theatrical body language was recommended. Additionally, the communication partner should align themselves at eye level with Ava and mirror her facial expressions. This will give Ava the impression that the receiver understands and is interested in communicating with Ava, and may also provide comfort, reassurance and build trust.

The ‘5S communication technique’ (i.e. Smile, Slow, Simple, Specific and Show) was recommended and explained.^57^ *Smiling* creates trust and reassurance. *Slow* speech allows time for Ava to process the message and to formulate a response. Communications should be *simple*, with one, short idea presented at a time, avoiding the use of slang or jargon, or asking more than one question at a time. Examples including mentioning *specific* people (using names instead of pronouns), objects and events familiar to her, and using gestures or visual cues such as photographs or pointing to items.

##### Personal care and ADLs

During personal care, Ava displayed resistive behaviour, including screaming and agitation. An individual with dementia may experience loss of control while receiving assisted personal care activities due to a lack of insight into their care needs and/or inability to communicate.^58^ Ava may also be sensitive to touch and temperature, so it is important to maintain a comfortable ambient temperature, ensure warm bathing water and provide adequate covering with towels. Ava was observed to respond well to having a female caregiver assisting, such as her mother, and enjoyed prompts for personal care with a handclap game prior to bathing. Assisting Ava to the bathroom slowly, with verbal and physical reassurance enables her to effectively process, adjust and connect to the activity.^58^

When preparing meals, Ava might benefit from observing cooking, or engaging in simple tasks such as sorting items, folding napkins or allowing her to touch and/or smell ingredients. Ava’s family should consider providing her with a relevant object such a cooking utensil to hold as she observes, to give her the sense of interaction and connection to the task. As Ava likes to bite things, it was suggested that Ava’s caregivers speak with a speech pathologist about gathering safe items for her to chew on.

Ava was reported to refuse medications at times. Her parents were advised to seek speech therapy support to assess her swallowing, and to continue regular visits as her condition progresses, in addition to discussing safe medication administration with the general practitioner (GP) and pharmacist.

To assist Ava with sensory changes, an occupational therapist can advise on making small adjustments around the house that may relieve fear during mobilisation, such as creating clearer passage by moving furniture, and fixing hand railings in appropriate locations around the house.

Ava’s cognitive impairment, together with her limited capacity to express her needs and desires verbally or physically, hyperacusis and physical pain are likely to be some of the multifactorial triggers of her BPSD.

##### Pain management

Ava’s parents informed the consultant that they believed Ava was not experiencing pain, as she was not expressing discomfort. However, using the Abbey Pain Scale,^59^ the agency caregivers reported severe pain for Ava likely due to neck stiffness and malformation. Non-verbal signs of pain observed included a tensed facial expression and screaming and agitation when mobilising. The consultant explained that as a result of her cognitive changes, Ava may no longer be able to express pain verbally, and that pain may instead manifest as agitation or screaming. Ava responded well to evening massages and PRN (pro re nata) paracetamol for neck pain. It was suggested that a GP should also be consulted about ongoing pain management.

#### Follow-up and outcomes

At follow-up 12 weeks later, there had been a significant reduction in NPI-Q total domain, distress and severity scores (Table 2).

The family reported that Ava had remained stable, and she was improved following the implementation of the consultant recommendations. Music and the sensory-stimulating items provided in the ‘private room’ were reported to have the most significant effects on her mood and behaviours. Ava responded well to evening relaxation music, massage and overall appeared to be more settled and less verbally aggressive when the ‘5S’ communication approach was used.^57^

Regular pain assessment was recommended, preferably by a clinical nurse specialised in pain management.

### Case 2: Bianca

#### Background

Bianca was a female with CLN3 Batten disease. Three months prior to the referral, Bianca had been moved from the family home into supported accommodation and was receiving ongoing support from a palliative care specialist, psychiatrist and behaviour support specialist.

Bianca was blind and presented with speech and language impairment, including loss of spoken expressive language, stuttering and dysarthria. As a result of extensive muscle spasms, myalgia, limb contractures and scoliosis, she was non-ambulatory and required hoisting. Bianca had developed a fear of falling, possibly as a result of an apparent deterioration in her balance and spatial awareness. She had also been experiencing an increase in seizures and aggressive behaviours.

#### Clinical findings

##### Behaviour and communication

Bianca was referred to DSA with the aim of providing support and assistance to accommodation staff, with Bianca’s increasing irritability and lability as the primary behaviours of concern. The consultant identified four probable contributing factors: loneliness/boredom, stress related to her recent change in living arrangement, caregiver approach and pain. Additionally, dementia education support was requested to enable care staff to better meet her needs through understanding Bianca’s CD condition and related behaviours.

The consultant observed Bianca to be quite vocal for most of the day. In the afternoon, after returning from her external day program, Bianca was often more distressed and agitated, screaming for up to 5 min. She was also frequently importunate, for example requesting music be turned on and off repeatedly. She would frequently perseverate before saying a full sentence and would repeatedly call out, increasingly louder.

Bianca appeared to experience difficulties with self-regulation when her attempts to communicate were missed, ignored or misunderstood, displaying persistence in her behaviour until her request(s) were addressed. She also appeared to experience hyperacusis and would frequently insist through echolalia that others could not speak except to her.

The consultant noted substantial environmental sound reverberation throughout the accommodation as a result of vinyl flooring and extensive flat, exposed surfaces.

##### Activities of daily living

Bianca required full assistance with ADLs. In recent months, she had developed moderate oropharyngeal dysphagia and required texture-modified foods and mildly thick fluids. She also experienced urinary incontinence and persistent constipation which appeared to cause pain.

She was observed to be particularly tired, typically falling asleep in the afternoons following day program activities.

#### Diagnostic assessment

Moderate-severe behaviours (Table 2) and moderate pain (PainChek^®^ score = 15) were recorded for Bianca. Bianca’s BPSD triggers were deemed multifactorial and included hunger/thirst, fatigue, over- or under-stimulation, excessive environmental noise, pain, an unnoticed absence seizure, urge incontinence, temperature dysregulation, medication change or adjustment to new routines.

#### Therapeutic interventions

##### Establishing routine and familiar environment

Given her blindness and cognitive decline, it is probable that Bianca had been experiencing distress since moving to a new environment in the supported group accommodation. Bianca may also experience under-stimulation and agitation or frustration in the absence of her usual routine, a structured environment and regular access to meaningful activities and enrichment. It was recommended that Bianca be given support and constant reassurance to help her adapt and become familiar with her new surroundings, and that this would also naturally require some time.

##### Sensory and meaningful engagement

Sensory engagement may assist with relaxation and reduce Bianca’s anxiety and agitation towards the end of each day.

A cloud-based voice service device was brokered to allow Bianca some autonomy over the music she listened to while providing access to information, audiobooks, podcasts, jokes and responses that could be tailored to her preferences and level of cognition. The robotic dog was brokered to assist Bianca with opportunities for engagement, amusement and comfort throughout the day, while the plush dog was provided to offer comfort throughout the night. Given Bianca’s vision impairment, her sense of touch was particularly important when engaging with her environment. Soft, textured fabrics such as velvet or looped fabrics to handle, and balls and small soft toys may be soothing. The yarn pack was intended to offer Bianca the option to roll up wool into balls, while also providing sensory engagement and the opportunity to do something meaningful. The Velcro game was chosen to engage Bianca’s sense of fun and humour as it allowed her to throw soft objects at a Velcro board and listen as they hit it. The bowl stabiliser was brokered so that Bianca could be involved in food preparation activities in the kitchen.

As Bianca becomes more familiar with caregivers, a scented foot or hand massage in the afternoon was suggested to help her to feel calm and safe. The consultant also discussed the soothing benefits of using a weighted blanket, in consultation with an occupational therapist.

##### Reducing overstimulation

To prevent or minimise irritation, lability and exhaustion, particularly given Bianca’s apparent hyperacusis, it was recommended that daily activities be tailored to reduce the length and intensity of her day program activities, including minimising noise and the presence of others. Arrangements were made for Bianca to travel home alone rather than with the group after day program activities.

When returning to her room, the consultant noted that the music offered to Bianca was quite loud. It was recommended that staff attempt reducing the volume, to assess if this is helpful in reducing her vocalisation behaviours.

To address fatigue, an afternoon quiet time was suggested, where Bianca could engage in a quiet, calming 1:1 activity, away from noisy, communal areas.

##### Distraction techniques

During times of anxious and fearful behaviour, or where known triggers such as loud noises may be present, Bianca might benefit from being moved to a quiet calm space, or may respond to distraction, for example, shifted to another topic or enjoyable activity. Care staff mentioned activities that appeared to decrease Bianca’s distress, including eating her favourite food, using humour and listening to music at low volume. Offering gentle verbal and physical reassurance, while maintaining a calm tone was also recommended.

##### Self-care

Staff reported that Bianca enjoys participating in hygiene and personal care activities. Engaging Bianca in her care will likely provide her with a sense of accomplishment. Bianca was more responsive to personal care if one caregiver-led communications and explained the care process step-by-step.

##### Communication techniques

The consultant explained that given her blindness, hyperacusis and cognitive and communicative changes, it may be challenging or distressing for Bianca to process and ‘filter’ when multiple people are talking to or around her. It was recommended that the care team should only have one person communicating with Bianca at a time, and that a calm, clear and reassuring voice should be used.

The ‘5S communication technique’ was recommended, as described in Ava’s referral above.^57^

To assist Bianca in orientating herself, it was recommended that staff continue to use Bianca’s name and speak directly with her, not in the third person, and to let her know when concluding a conversation.

In addition, a ‘conversation box’ created by her family was left in Bianca’s room, containing items staff could use to engage with Bianca, including talking photo albums. Staff had reportedly been incorporating the box of items into Bianca’s daily routine, which had been helpful.

##### Pain management

If Bianca is agitated or distressed, it was recommended that staff should investigate organic factors such as pain, constipation, incontinence, fatigue, muscle stiffness or discomfort related to menstruation. A regular non-verbal pain assessment, for example, Abbey Pain Scale^59^ or PainChek^45^ might help staff identify whether Bianca is experiencing pain at specific times. Given her speech and language difficulties, it may not always be possible for Bianca to report her experience of pain and/or discomfort, and this may be expressed through changes in mood, body language, increased agitation, repetitiveness or vocalisation.

##### Family and caregiver support

Bianca’s family was informed about support groups and 1:1 counselling that may be beneficial for dealing with the significant changes in Bianca’s life. The DSA consultant also identified that there was a need for staff support and education around CD as some caregivers were having difficulty distinguishing and managing BPSD.

Bianca’s care team were provided with information resources on managing hallucinations and delusions, medication management issues, the use of music engagement in dementia care, validation techniques and reminiscence, understanding the impact of pain in dementia and providing personalised care for a person living with dementia.

Care staff were encouraged to continue with detailed behaviour charts and documenting experiences before and after care to allow the care team to identify helpful means of settling Bianca. The consultant suggested creating a ‘10 facts about me’ information sheet for Bianca to distribute amongst caring staff. The DSA consultant provided educational sessions as part of the support episode and also recommended for the home to arrange an external agency to provide ongoing dementia education sessions for the staff.

### Follow-up and outcomes

At follow-up 16 weeks later, there had been a reduction in the NPI-Q total domain, distress and severity scores (Table 2). Bianca’s behaviour had improved with the implementation of a routine, including the use of a whiteboard to display her daily schedule. Her distress had also noticeably reduced when care staff provided one-on-one personal care.

Dementia training for care staff had been well received. Staff had gained greater confidence and understanding of BPSD and in addressing personal needs of Bianca. As a result, the home manager felt comfortable closing the referral with DSA.

## Discussion and implications for care

We discussed two CD cases with BPSD presentation, where multimodal person-centred psychosocial interventions were provided by a dementia-specific behaviour support service. To our knowledge, these are the first published case reports of CD individuals with BPSD, supported by the same dedicated external service.

Our key findings suggest that multimodal person-centred psychosocial interventions may be useful or effective in managing BPSD in individuals with CD, consistent with guiding standards and principles of supportive and palliative care for Sanfilippo syndrome and Batten disease.^27,60–62^ These interventions entail maintaining quality of life, capacity and independence for as long as possible with an emphasis on holistic, multidisciplinary care, symptom management and ongoing support for both the person and their family unit. Importantly, the management of BPSD requires an understanding of the behaviours in the context of the cognitive skill level and should evolve over time with a growing emphasis on modifying the environment and expectations, rather than on ‘training’ the affected individual.^27,62^

Psychosocial impacts of CD on affected individuals and their families have been discussed in the literature; however, formal evaluation of multimodal psychosocial interventions for CD remain extremely limited.^16,18,30,63–65^ Such interventions, however, are considered first-line management therapies for adult-onset dementia.^66,67^ With the support of dementia-specific consultants, the two cases reported here show improvement across neuropsychiatric outcomes of the NPI-Q and may highlight the effectiveness and importance of multimodal psychosocial support when dealing with complex behavioural problems in individuals with these conditions.^18,19^ The evidence corroborates the appointment of dementia specialists, person-centred case management and educational sessions for positive psychosocial outcomes for individuals with dementia.^65,68^

### Living environment and routine

In both cases, consultants provided recommendations and supported caregivers to create safe, familiar environment by introducing or modifying certain items, practices and routines. Clinical guidelines for the management of Sanfilippo syndrome and Batten disease recommend establishing routines and increasing familiarity with the environments to minimise triggering behaviours.^62,69–71^ Routine has been associated with a reduction in aggression, agitation and anxiety in individuals with dementia.^30,69–71^

### Meaningful engagement and sensory stimulation

Sensory stimulation aims to prevent sensory deprivation in individuals with cognitive decline by creating an enriched environment.^72^ Sensory stimulation interventions are known to increase alertness, reduce agitation and improve quality of life in older adults with dementia.^73^ Sensory items including activity blankets had a positive impact on Ava’s behaviour. These blankets are used as a sensory engagement intervention for individuals with dementia who are agitated or anxious, and is designed to trigger positive memories, provide multisensory stimulation and stress relief, and to keep ‘restless hands’ busy through distraction.^74^

Music therapy/engagement has been demonstrated to improve cognitive performance, including^78^ access to memories in individuals CLN3 disease and Sanfilippo syndrome.^67,75,76^ It has also been shown to improve communication and quality of life and reduce the incidence of aggressive behaviour in adults with dementia.^77^

Participation in ADLs has been recommended to maintain skills and quality of life, particularly in the later stages of CD disease progression.^64^ In both cases, caregivers were advised to encourage person involvement in meal preparation by engaging in simple tasks such as sorting items and folding napkins.

### Reducing overstimulation

Ava and Bianca presented with anxious behaviours, aggression and frustration/irritability. Studies demonstrate that overstimulation, and stressful life events such as relocation, as experienced by Bianca, are associated with an increase in aggressive behaviours and negative health outcomes such as depression and anxiety.^69,71,78,79^

Reducing exposure to negative stimuli was beneficial in both reported cases. DSA consultants recommended that ADLs are tailored to the individual’s current level of cognition, including the provision of quiet one-on-one activities, cessation of behaviour-induced activities, avoidance of crowded, loud environments (e.g. avoidance of multiple people speaking at once) and caregivers use of a calm and reassuring tone of voice.^62^ Non-pharmacological approaches to hyperactivity and anxious behaviours, such as establishing routine, singing and ‘busying’ the child, have been reported to be highly effective.^80^

### Communication techniques

For both cases, positive communication including the ‘5S communication technique’ was recommended and explained to caregivers.^57^ In both cases, there was a reduction in agitation and verbal aggression including screaming out and repetitive vocalisations, along with a reduction in physical aggression observed in Ava.

Progressive decline in speech, language and communication are common features of CDs and may be a contributing factor to the anxious and irritable behaviours observed in these conditions.^12,16,19,30^ Speech therapy has been identified as an essential component in holistic multidisciplinary management in Sanfilippo syndrome and Batten disease.^27,60,61^

Techniques like reminiscence take place in the present while establishing a connection to the past, serving as a form of distraction and soothing therapy, enabling individuals with dementia to maintain a sense of continuity in their identity and restore communication.^81,82^ In addition to reminiscent techniques, validation therapy was also suggested, wherein caregivers are guided to respond to the affected individual’s expressed feelings, and to validate those feelings and the individual’s perceived reality.^82^ These alternative communication approaches have been beneficial interventions for individuals with adult-onset dementia, although there has been extremely limited research in the context of CD.^83^

### Pain management

Pain was identified as a contributing factor to BPSD in both Ava and Bianca, ranging from moderate to severe pain. Individuals with CD are more likely to experience frequent or persistent pain compared to their cognitively intact counterparts, due to associated conditions including movement disorders such as dystonia and spasticity, seizures, constipation and other gastrointestinal complications, scoliosis, self-injurious behaviour and infections (e.g. urinary tract).^27,62,84–87^ As evident in both cases, due to the decline in language and communication skills, and progressive cognitive impairment in CD, individuals may be unable to self-report pain.^86,87^ As such, management guidelines recommend regular pain assessments to be part of ongoing care and management plans for optimal pain identification and management.^27,62,84,86,87^

### Limitations

This study has some limitations that relate to the rarity of CD. It is difficult to sample participants with CD given the epidemiology of these disorders. As such, the findings were not a direct comparison between the cases. Another limitation was the use of neuropsychiatric and pain assessment tools to measure these clinical variables in our study. These measures were not specifically validated and may not be appropriate for use in this population. Future research should target the validity and reliability of these tools for children and adolescents with dementia.

It is important to note that the clinical presentations of both Batten disease and Sanfilippo syndrome are heterogeneous, not only between subtypes of these conditions, but also between individuals with the same subtype. Consequently, the process of evaluating individual cases and determining effective management strategies can be fraught with significant complexities.

The effectiveness of psychosocial interventions may be influenced by cognitive capacity and symptom severity, emphasising the importance of a person-centred care plan for of BPSD.^88^ In cases with impaired cognition, such as those presented here, targeted and timely multimodal psychosocial interventions are necessary. To capture the information of interest, a qualitative descriptive and narrative approach was considered the most suitable. A detailed analysis and observation of the cases has provided a rich source of qualitative information regarding the nature and extent of the BPSD exhibited by these individuals and the solutions offered by external support agencies. While there are some similarities between clinical presentations of Batten disease and Sanfilippo syndrome, there are also significant differences in symptomatology and underlying aetiologies, such that varied responses to the same psychosocial interventions are to be expected. We therefore applied a case study methodology to account for the exploratory nature of this investigation.

Some follow-up reports were provided by means of caregiver interview. Given the subjectivity of such reports, we must exercise caution in interpretation of the contribution each individual intervention recommendation made in effectuating a reduction in the referrals’ NPI-Q outcomes. Furthermore, it is possible that insufficient time had passed between the implementation of consultant recommendations and follow-up. To this point however, we must also be simultaneously mindful that persistent neurological deterioration is a feature of these conditions, particularly in the later stages, and therefore, apparent ineffectiveness of recommended interventions may not be a result of inadequate management, but a result of the underlying changes or progression of the disease over this period. Since DSA support is episodic in nature (typically 2–3 months on average), there is limited follow-up and longitudinal data that can be obtained for the cases. Finally, given the notable lack of existing research on psychosocial interventions in the context of CD, we are unable to draw comparisons with our findings.

## Conclusion

These cases demonstrate that multimodal, person-centred psychosocial care delivered through national dementia-specific support programs may help alleviate BPSD associated with the CDs Batten disease and Sanfilippo syndrome. Consultants introduced resources and strategies through multimodal interventions that were reported to positively impact the quality of life of these individuals and their caregivers. Modifying caregiver approach and increasing CD-specific education around dementia and related communication changes were particularly beneficial in the management of BPSD in both cases. The success of the interventions may also be attributed to close collaboration between DSA consultants and peak bodies BDSRA Australia and Childhood Dementia Initiative. This highlights the importance of collaborative efforts with peak bodies who may provide the necessary disease-state knowledge and expertise, to complement DSA’s professional experience with BPSD management and interventions.

## Supplemental Material

sj-pdf-1-pcr-10.1177_26323524241273492 – Supplemental material for Behaviours and psychological symptoms of childhood dementia: two cases of psychosocial interventions Supplemental material, sj-pdf-1-pcr-10.1177_26323524241273492 for Behaviours and psychological symptoms of childhood dementia: two cases of psychosocial interventions by Mustafa Atee, Ineka Whiteman, Rebecca Lloyd and Thomas Morris in Palliative Care and Social Practice

## Declarations

**Ethics approval and consent to participate:** The study received ethical approvals with a waiver of informed consent from Human Research Ethics Committee of each of the University of New South Wales (UNSW; HC190049), Curtin University (HRE2023-0069) and the University of Sydney (2023/138). Study data were de-identified and handled by a data custodian who complied with the Declaration of Helsinki of ethical principles, and the National Health and Medical Research Council’s National Statement on Ethical Conduct in Human Research (2018). To preserve anonymity and confidentiality of reported cases, all identifying information (e.g. exact age, case length) have been removed from the case report materials due to the possibility of easily identifying such information for individuals with rare disorders.

**Consent for publication:** A waiver of informed consent from patients was granted as per the ethics approvals stated above.

**Competing interests:** MA is one of the originators of the PainChek^®^ instrument, which is marketed by PainChek Ltd. (ASX: PCK). He is also a shareholder of PainChek Ltd. He previously held the position of a Senior Research Scientist (October 2018–May 2020) at PainChek Ltd. He had a granted patent titled ‘A pain assessment method and system; PCT/AU2015/000501’ in China, Japan, the European Union and the United States, which was assigned to PainChek Ltd. Three authors (MA, TM, RL) are staff members of The Dementia Centre; a research, education and consultancy arm of HammondCare, an independent Christian charity which auspices the DSA programs. MA is a Research and Practice Lead (Team Leader) at The Dementia Centre, HammondCare. RL is a Data and Information Analyst at The Dementia Centre, HammondCare. TM is Head of Research and Information Excellence, The Dementia Centre, HammondCare.

**Availability of data and materials:** The cases were anonymised for ethical and privacy reasons and also to comply with the conditions of service provision of Dementia Support Australia (DSA).
