# Corrigendum to “Behaviours and psychological symptoms of childhood dementia: two cases of psychosocial interventions”

**DOI:** 10.1177/26323524251361081

**Published:** 2025-07-22

Atee M, Whiteman I, Lloyd R, Morris T. Behaviours and psychological symptoms of childhood dementia: two cases of psychosocial interventions. *Palliative Care and Social Practice*. 2024;18. doi:10.1177/26323524241273492

The following corrections are made in the above-referenced article:

- In Table 2, the third column, the medication profile for Ava incorrectly includes “cannabis oil for agitation management” and “atropine drops for hypersalivation”, so these medications are deleted.
- In Table 2, the third column, the medication profile for Bianca incorrectly includes “macrogol (PRN) for constipation relief” and “sodium valproate tablets as a mood stabiliser”. The latter should be read as “sodium valproate and clobazam tablets for seizure management”.
- Under “Therapeutic interventions,” 'supported group accommodation' is changed to 'supported accommodation’.

The journal editor confirms that these changes do not alter the conclusions.

The authors apologise to readers for these inadvertent errors.

