# Supplementary material for: Behaviours and psychological symptoms of childhood dementia: two cases of psychosocial interventions
Source: Palliat Care Soc Pract. 2024 Sep 6;18:26323524241273492. doi: 10.1177/26323524241273492 (PMC11378187; doi:10.1177/26323524241273492)
Supplement: sj-pdf-1-pcr-10.1177_26323524241273492 – Supplemental material for Behaviours and psychological symptoms of childhood dementia: two cases of psychosocial interventions [file sj-pdf-1-pcr-10.1177_26323524241273492.pdf]

CARE case report guidelines

CARE Checklist of information to include when writing a case report

(cc) BY-NC-ND

Topic	Item	Checklist item description	Reported on Line
Title	1	The diagnosis or intervention of primary focus followed by the words "case report"	1 - 2
Key Words	2	2 to 5 key words that identify diagnoses or interventions in this case report, including "case report"	28 -29
Abstract (no references)	3a	Introduction: What is unique about this case and what does it add to the scientific literature?	6 - 16, 20 - 25
	3b	Main symptoms and/or important clinical findings	16 - 21
	3c	The main diagnoses, therapeutic interventions, and outcomes	8 - 10, 12 - 16,16 - 21
	3d	Conclusion—What is the main "take-away" lesson(s) from this case?	20 - 25
Introduction	4	One or two paragraphs summarizing why this case is unique (**may include references**)	100 - 114
Patient Information	5a	De-identified patient specific information.	166, 590 - 592
	5b	Primary concerns and symptoms of the patient.	Case 1: 189 - 219, Case 2: 317 - 350
	5c	Medical, family, and psycho-social history including relevant genetic information	Table 2, Case 1: 189 - 195, Case 2: 317 - 324
	5d	Relevant past interventions with outcomes	100 - 114
Clinical Findings	6	Describe significant physical examination (PE) and important clinical findings.	Case 1: 192 - 195, 198 - 219, Case 2: 320 - 324, 328- 350
Timeline	7	Historical and current information from this episode of care organized as a timeline	172 - 173, Case 1: 189 - 195, 305 - 313, Case 2: 317 - 324, 442 - 448
Diagnostic Assessment	8a	Diagnostic testing (such as PE, laboratory testing, imaging, surveys).	Tables 1 and 2, 117 - 163
	8b	Diagnostic challenges (such as access to testing, financial, or cultural)	535 - 567
	8c	Diagnosis (including other diagnoses considered)	Tables 1 and 2, 117 - 163, Case 1: 189 - 195, Case 2: 317 - 324
	8d	Prognosis (such as staging in oncology) where applicable	Case 1: 73- 80, Case 2: 87 - 91
Therapeutic Intervention	9a	Types of therapeutic intervention (such as pharmacologic, surgical, preventive, self-care)	Table 2, Case 1: 226 - 302, Case 2: 360 - 439
	9b	Administration of therapeutic intervention (such as dosage, strength, duration)	117 - 163, 172 - 173, 460 - 463, 535 - 567, Case 1: 228 - 230, Case 2: 363 - 365
	9c	Changes in therapeutic intervention (with rationale)	N/A
Follow-up and Outcomes	10a	Clinician and patient-assessed outcomes (if available)	467 - 470, 525 - 533, Table 2, Case 1: 305 - 313, Case 2: 442 - 448
	10b	Important follow-up diagnostic and other test results	Table 2, Case 1: 305 - 313, Case 2: 442 - 448
	10c	Intervention adherence and tolerability (How was this assessed?)	535 - 567, Case 1: 305 - 313, Case 2: 442 - 448
	10d	Adverse and unanticipated events	N/A
Discussion	11a	A scientific discussion of the strengths AND limitations associated with this case report	451 - 472, 535 - 567
	11b	Discussion of the relevant medical literature **with references**.	451 - 533
	11c	The scientific rationale for any conclusions (including assessment of possible causes)	451 - 580
	11d	The primary "take-away" lessons of this case report (without references) in a one paragraph conclusion	570 - 580
Patient Perspective	12	The patient should share their perspective in one to two paragraphs on the treatment(s) they received	Case 1: 305 - 313, Case 2: 442 - 448
Informed Consent	13	Did the patient give informed consent? Please provide if requested Waived- consent as per ethics approval	Yes ☐ No ☑
